# A new species of the genus *Timalinyssus* Mironov, 2001 (Acarina, Psoroptidia) with a key to known species

**DOI:** 10.3897/zookeys.557.7098

**Published:** 2016-01-28

**Authors:** Ioana Cristina Constantinescu, Gabriel Chişamera, D. Khlur B. Mukhim, Costică Adam

**Affiliations:** 1 “Grigore Antipa” National Museum of Natural History, Sos. Kiseleff no.1, 011341 Bucharest, Romania; 2 Zoology Department, Lady Keane College, 793001 Shillong, Meghalaya, India

**Keywords:** Pteronyssidae, Timalinyssus wahlangi, new species, systematics

## Abstract

The article describes a new species of the feather mite family Pteronyssidae (Acarina: Psoroptidia) from the Gray Sibia *Heterophasia gracilis* (McClelland) (Passeriformes, Leiothrichidae) in India (Meghalaya, Jaintia Hills, Shnongrim village). Males of *Timalinyssus wahlangi* **sp. n.** differ from those of all *Timalinyssus* species by having the horseshoe-shaped epiandrum with a short anterior extension. Females of the new species differ from those of all previously known species of the genus in having the hysteronotal shield with deep lateral incisions between *e2* and *f2* setae. A key to all species of the genus *Timalinyssus* is presented.

## Introduction

The feather mite family Pteronyssidae currently includes about 180 species in 23 genera (Gaud and Mouchet 1959; Faccini and Atyeo 1981, Mironov 2001, 2005; Mironov and Wauthy 2005a, 2005b, 2008; Mironov and Proctor 2011; Constantinescu et al. 2014a, 2014b). Within this family, the genus *Timalinyssus* Mironov encompasses six species of large-sized mites that can be found on birds of the families Leiothrichidae and Paradoxornithidae (Passeriformes) from Asia (China, Taiwan, Vietnam and India). The type species is *Timalinyssus formosanus* Mironov, 2001 from *Actinodura morrisoniana* (Ogilvie-Grant). Initial diagnostic characters given to the genus (Mironov 2001) proved to be insufficient as new species were subsequently described (Wang and Wang 2008; Mironov and Proctor 2011; Constantinescu et al. 2014a). Mironov and Proctor (2011) described the distinctive feature differentiating it from the closely related genus *Mouchetia*, namely the structure of tarsus III in males. In *Timalinyssus*, tarsus III is usually elongated and curved, with a claw-like or bidentate apical process and the dorsal surface of this segment bearing a smooth or indented longitudinal crest (in the case of *Timalinyssus oliferae*, the longitudinal crest is absent but one rounded dorsal tooth is present). Males of *Mouchetia* have tarsus III with a large spine on apex and subapical spine on the outer margin of this segment. Females of *Timalinyssus* differ from those of the genus *Mouchetia* in having the hysteronotal shield not narrowed in the anterior half. In the present paper a new *Timalinyssus* species found on the Gray Sibia *Heterophasia gracilis* (McClelland) is described and a key to all known species of the genus is also provided.

## Materials and methods

The material used in the present paper was collected in Meghalaya (India) in January 2014. The birds were captured using mist-nets, identified and visually checked for the presence of mites and after collecting them released back into the wild. Mite specimens were taken from birds manually with a needle and placed in vials with ethanol. Later, in the laboratory, the mite specimens were cleared in lactic acid and mounted on microscope slides in Hoyer’s medium. Drawings were made using an Olympus CX21 microscope, with a camera lucida drawing device. The bird specimens were identified according to Rasmussen and Anderton (2012) and Grimmett et al. (2011), and the taxonomy of the birds follows Clements et al. (2013). The setation of mite’s body follows that of Griffiths et al. (1990) with modifications of Norton (1998) concerning coxal setae, while the setation of legs follows Gaud and Atyeo (1996). The description of *Timalinyssus wahlangi* sp. n. is given according to the current format used for species of the pteronyssid taxa (Faccini and Atyeo 1981; Hernandes 2012; Mironov 1992, 2001). The measuring techniques of particular structures used in the present paper were described by Mironov and Proctor (2011). We give the full set of measurements for a holotype (male) and range of measurements for corresponding paratypes. All measurements are in micrometres (μm). The holotypes and all paratypes of the new species are deposited in the Acarological Collection of the “Grigore Antipa” National Museum of Natural History, Bucharest, Romania.

## Results

### Family Pteronyssidae Oudemans, 1941 Genus *Timalinyssus* Mironov, 2001

#### Timalinyssus wahlangi sp. n.

Taxon classificationAnimaliaAstigmataPteronyssidae

http://zoobank.org/7E0C381A-DEC3-47D8-90F8-7FB83F2FC293

**Figure 1.**
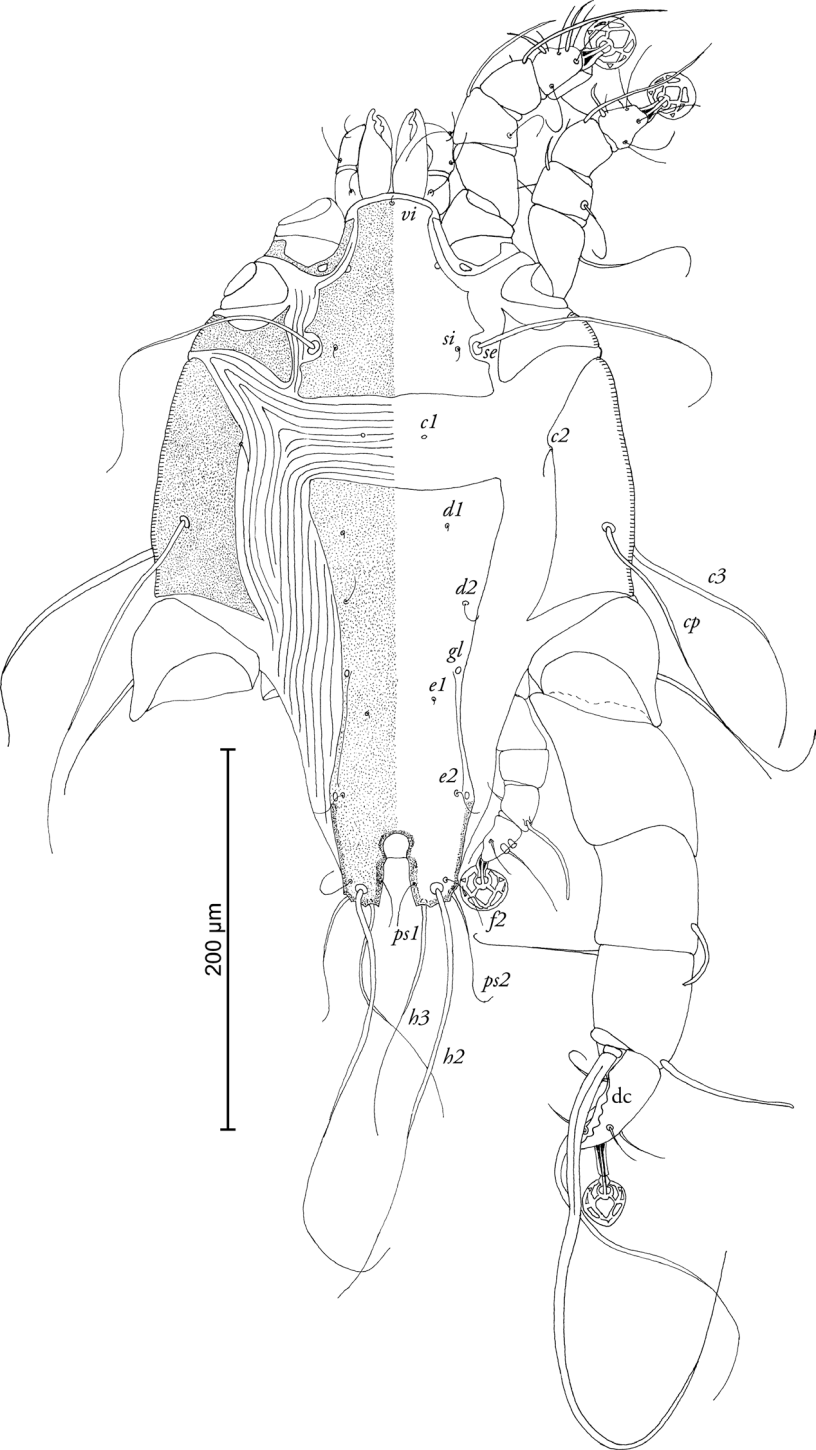
*Timalinyssus wahlangi* sp. n., male holotype: dorsal view of idiosoma. Abbreviations: dc – dorsal crest.

**Figure 2.**
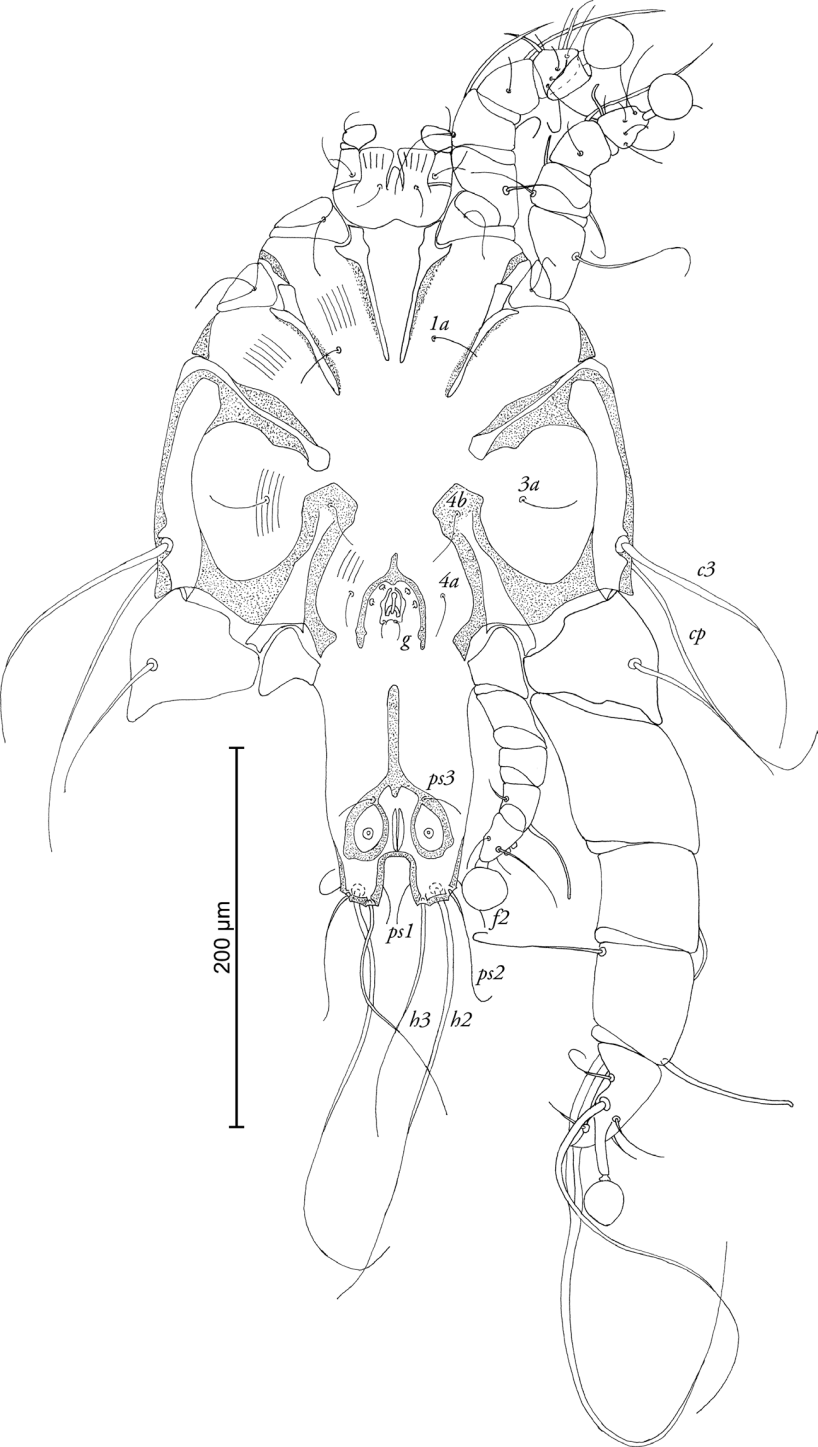
*Timalinyssus wahlangi* sp. n., male holotype: ventral view of idiosoma.

**Figure 3.**
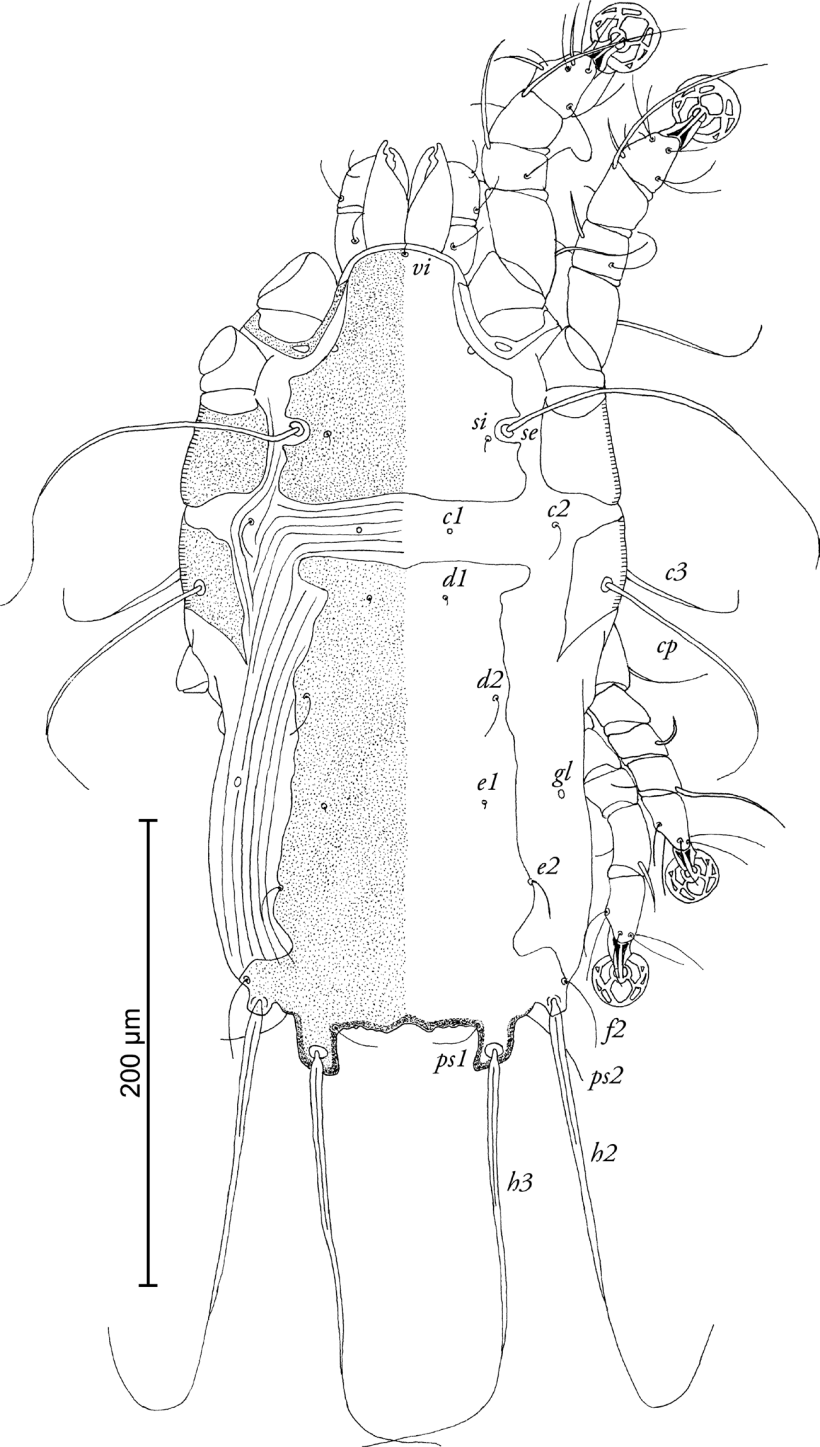
*Timalinyssus wahlangi* sp. n., female paratype: dorsal view of idiosoma.

**Figure 4.**
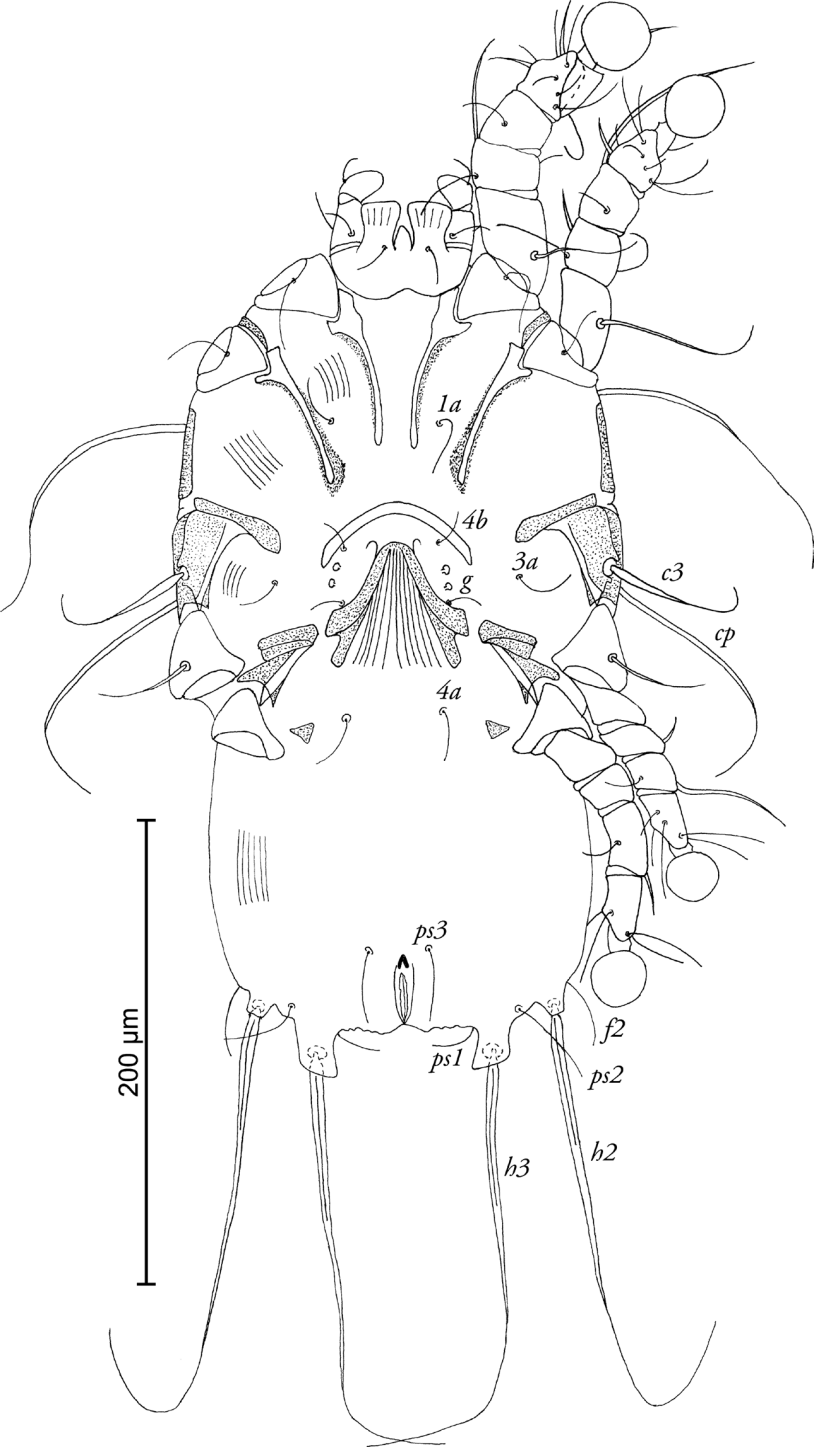
*Timalinyssus wahlangi* sp. n., female paratype: ventral view of idiosoma.

**Figure 5.**
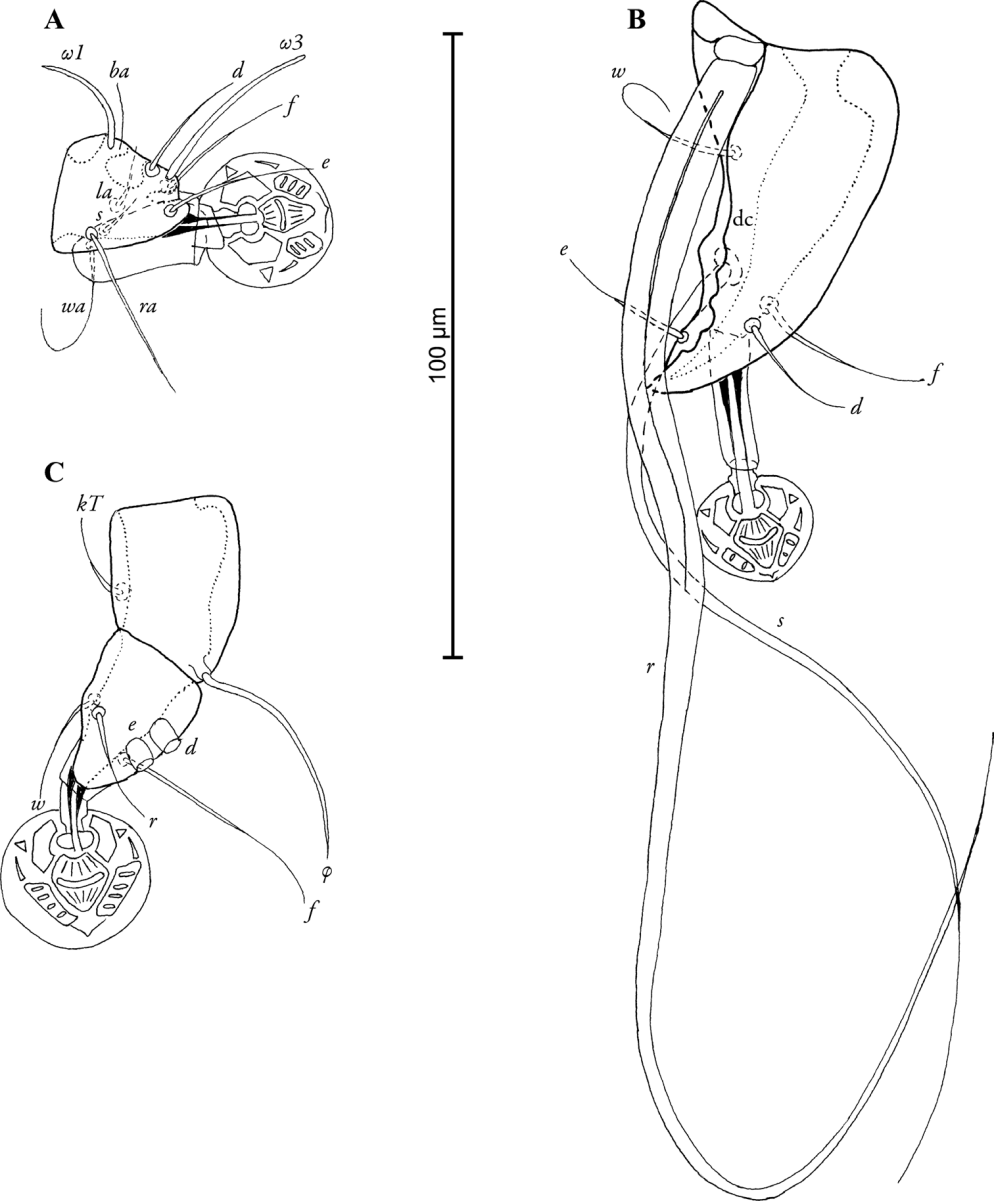
*Timalinyssus wahlangi* sp. n., details of male legs, dorsal view: **A** tarsus of leg I **B** tarsus of leg III **C** tibia and tarsus of leg IV; Abbreviations: dc – dorsal crest.

Figs 1 , 2 , 3 , 4 , 5

##### Type material.

Male holotype (ANA450), 3 male (ANA448, ANA449, ANA451) and 1 female (ANA452) paratypes 25.01.2014, 3 female (ANA445, ANA446, ANA447) paratypes 20.01.2014, from the Gray Sibia *Heterophasia gracilis* (McClelland) (Passeriformes, Leiothrichidae); **INDIA**: Meghalaya, Jaintia Hills, Shnongrim village, (25°21'12.36"N, 92°31'3.06"E); 1151 m; subtropical forest; collector D. Khlur B. Mukhim.

##### Description.

MALE (Figs 1; 2; 5A–C; holotype, range for 3 paratypes in parantheses): Idiosoma 370 long (370–380), 250 wide (240–260). Prodorsal shield length 100 (100–110), width 92 (92–98), not fused with scapular shields. Distance between bases of setae *se* 80 (80–82), distance between bases of setae *si* 62 (60–63), posterior margin almost straight, lateral margins with small incisions at level of setae *se.* Setae *c2* short, filiform, about 20 (15–20) in length, situated on medial margins of humeral shields. Setae *c3* enlarged in basal part and filiform in apical part, 130 (125–140) in length. Hysteronotal shield with slightly concave anterior margin, anterior angles rounded, length 220 (215–230), width at anterior margin 100 (98–105). Distance along midline between prodorsal and hysteronotal shields 44 (44–56). Width of opisthosoma at level of setae *f2* 58 (58–66). Opisthosomal lobes short, with acute inner and lateral angles and bidentate posterior margin. Terminal cleft U-shaped, length 20 (18–21), supranal concavity opens posteriorly. Position of setae *e1* posterior to gland openings *gl.* Lengths of dorsal setae: *c2-d2* 94 (94–105), *d2-e2* 98 (98–110), *d2-gl* 36 (36–37), *e2-h2* 46 (36–46), *h2-h2* 42 (42–54), *h3-h3* 28 (28–30), *ps1-h3* 8 (6–8). Transventral sclerite absent, epiandrum horseshoe-shaped with short anterior extension, posterior tips extending considerably beyond base of genital apparatus (Fig. 2). Length of genital apparatus 18 (18–20), width at base 12 (12–14), aedeagus length 12 (9–12). Setae *g* situated on base of genital arch. Anal suckers ovate, their size excluding surrounding membrane: longer diameter 18 (14–18), shorter diameter 12 (12–14). Adanal shield shaped as an inverted Y, narrow, almost completely encircling anal field. Ventral measurements: *4b-3a* 38 (38–40), *4a-g* 24 (22–26), *3a-4a* 68 (68–76), *ps3-ps3* 26 (20–26), *ps3-h3* 44 (42–44). Tarsus III 60 (58–74) in length, with acute apical process and 5 denticles on dorsal longitudinal crest, macrochaeta *r* with very thick basal part, macrochaeta *s* about 1/3 of macrochaeta *r*, other tarsal setae filiform, shorter than segment (Fig. 5 B).

**Table 1.** *Timalinyssus* species and their host associations.

Mite species	Host species	Host family	Location	References
*Timalinyssus oliferae* (Mironov, 1990)	*Leiothrix argentauris* (Hodgson)	Leiothrichidae	Vietnam	Mironov 1990; Mironov 2001
*Timalinyssus formosanus* Mironov, 2001	*Actinodura morrisoniana* Ogilvie-Grant	Leiothrichidae	Taiwan	Mironov 2001
*Timalinyssus longitarsus* Wang & Wang, 2008	*Garrulax canorus canorus* (Linnaeus)	Leiothrichidae	China	Wang and Wang 2008
	*Garrulax pectoralis* (Gould)	Leiothrichidae	China	Mironov and Proctor 2011
*Timalinyssus curvilobus* Mironov & Proctor, 2011	*Ianthocincla sannio* (Swinhoe)	Paradoxornithidae	China	Mironov and Proctor 2011
*Timalinyssus grallator* Mironov & Proctor, 2011	*Lioparus chrysotis* (Blyth)	Leiothrichidae	China	Mironov and Proctor 2011
*Timalinyssus actinodurae* Constantinescu, 2014	*Actinodura cyanouroptera* (Hodgson)	Leiothrichidae	India	Constantinescu et al. 2014a
*Timalinyssus wahlangi* sp. n.	*Heterophasia gracilis* (McClelland)	Leiothrichidae	India	Present paper

FEMALE (Figs 3; 4; range for 4 paratypes): Idiosoma 345–380 long, 185–200 wide. Prodorsal shield not fused with scapular shields, posterior margin slightly concave, length of shield 98–100, width 100-110, setae *se* separated by 82–92. Setae *c2* hair-like, about 12–14 long, situated on striated tegument. Hysteronotal shield almost rectangular, with anterior margin slightly concave, anterior part of this shield with rounded lateral extensions, lateral margins with deep incisions between bases of setae *e2* and *f2*, length 220–230, width at anterior margin 96–110. Distance along midline between prodorsal and hysteronotal shields 28–36. Posterior end of opisthosoma with 1 pair of widely separated opisthosomal lobes bearing bases of setae *h3*. Opisthosomal lobes small, with oblique posterior margin, without membrane. Length of terminal cleft 18–24, width at lobar bases 52–68. Position of setae *e1* posterior to gland openings *gl.* Dorsal measurements *c2-d2* 74–82, *d2-e2* 80–90, *e2-h3* 72–74, *d2-gl* 40–44, *e1-gl* 30–40, *h2-ps1* 34–38, *h2-h2* 110–120, *h3-h3* 70–88. Epigynium approximately semicircular, 28–30 long, 66–72 wide. Apodemes of egg-laying opening extending to midlevel of trochanters III. Epimerites IVa present, rudimentary. Legs IV extending to level of setae *h2*.

##### Etymology.

The new species is named in a memory of Mr. Dran Wahlang, a father of the junior coauthor, D. Khlur B. Mukhim.

##### Remarks.

Of the six previously known species, *Timalinyssus wahlangi* sp. n. is closest to *Timalinyssus actinodurae* Constantinescu, 2014 from *Actinodura cyanouroptera* (Hodgson) (Leiothrichidae) (Constantinescu et al. 2014a). Males in both species have the prodorsal shield not fused with scapular shields, setae *c2* situated on medial margins of the humeral shields, the adanal shield shaped as an inverted Y, a similar shape of tarsus III with an acute apical process and small denticles on the dorsal longitudinal crest, and setae *r* and *s* represented by macrochaetae. Males of the new species clearly differ from those of *Timalinyssus actinodurae* in having the following features: setae *se* are situated on the striated tegument, setae *e1* are situated posterior to the level of gland openings *gl*, the hysteronotal shield has a concave anterior margin, setae *ps1* are situated clearly distant from the inner angle of the opisthosomal lobes, the transventral sclerite is absent, the epiandrum is horseshoe-shaped with short anterior extension, and dorsal longitudinal crest of tarsus III has 4-5 denticles. In males of *Timalinyssus actinodurae*, setae *se* are situated on the prodorsal shield, setae *e1* are situated approximately at the same transverse level with the gland openings *gl*, the hysteronotal shield has a straight anterior margin, the setae *ps1* are situated almost apically, the transventral sclerite is present, epiandrum is shaped as an inverted U and fused with the posterior end of the transventral sclerite, and the dorsal longitudinal crest of tarsus III has 2 denticles. Females in both species have opisthosomal lobes short and separated by wide terminal cleft and the hysteronotal shield with lateral extensions in anterior part. Females of *Timalinyssus wahlangi* sp. n. differ from those of *Timalinyssus actinodurae* (and also of the other five known species) by the shape of the hysteronotal shield that has lateral margins with deep incisions between setae *e2* and *f2*. Furthermore, females of the new species differ from those of *Timalinyssus actinodurae* in having the prodorsal shield not fused with scapular shields, setae *se* situated on the striated tegument, the opisthosomal lobes without lateral membranes, and legs IV extending to the level of setae *h2*. Females of *Timalinyssus actinodurae* have the prodorsal shield fused with the scapular shields, setae *se* are situated on the prodorsal shield, the opisthosomal lobes have lateral membranes, and legs IV do not extend to the level of setae *h2*.

### Key to males of *Timalinyssus*

**Table:** 

1	Prodorsal shield fused with scapular shields	**2**
–	Prodorsal shield not fused with scapular shields	**3**
2	Setae *se* situated on prodorsal shield, transventral sclerite present, setae *ps2* narrowly lanceolate, setae *h3* longer than *h2*, tarsus III with one macrochaeta *r*	***Timalinyssus oliferae***
–	Setae *se* situated on striated tegument, transventral sclerite absent, setae *ps2* filiform, setae *h2* longer than *h3*, tarsus III with two macrochaetae, *r* and *d*	***Timalinyssus formosanus***
3	Setae *c2* situated on medial margin of humeral shields	**4**
–	Setae *c2* situated on striated tegument or on anterior margin of humeral shields	**5**
4	Transventral sclerite present, setae *e1* and gland openings *gl* at the same transverse level, dorsal longitudinal crest of tarsus III with 2 poorly distinct denticles	***Timalinyssus actinodurae***
–	Transventral sclerite absent, setae *e1* situated posterior to level of gland openings *gl*, dorsal longitudinal crest of tarsus III with 4-5 denticles	***Timalinyssus wahlangi* sp. n.**
5	Setae *c2* situated on anterior margin of humeral shields, opisthosomal lobes strongly elongated and bifurcate apically, legs III longer then length of idiosoma	***Timalinyssus grallator***
–	Setae *c2* situated on striated tegument, opisthosomal lobes short and without apical bifurcation, legs III shorter then length of idiosoma	**6**
6	Opisthosomal lobes straight, epiandrum present; tarsus III with acute apical process, two macrochaetae *r* and s, and indented dorsal crest	***Timalinyssus longitarsus***
–	Opisthosomal lobes bent towards, epiandrum absent; tarsus III with bidentate apical process, one macrochaeta *r*, and smooth dorsal crest	***Timalinyssus curvilobus***

### Key to females of *Timalinyssus*

(Female of *Timalinyssus grallator* unknown)

**Table:** 

1	Dorsal setae *f2* and *h2* situated on hysteronotal shield	**2**
–	Dorsal setae *f2* and *h2* situated on striated tegument	***Timalinyssus longitarsus***
2	Opisthosomal lobes present, external copulatory tube absent	**3**
–	Without distinct opisthosomal lobes, external copulatory tube present	***Timalinyssus curvilobus***
3	Opisthosomal lobes longer than wide and separated by narrow terminal cleft	**4**
–	Opisthosomal lobes small and separated by terminal cleft much wider than lobes	**5**
4	Anterior part of hysteronotal shield with rounded lateral extensions, setae *e1* anterior to level of gland openings *gl*, setae *se* situated on prodorsal shield	***Timalinyssus oliferae***
–	Anterior part of hysteronotal shield without rounded lateral extensions, setae *e1* posterior to level of gland openings *gl*, setae *se* situated on striated tegument	***Timalinyssus formosanus***
5	Prodorsal shield fused with scapular shields, setae *se* on prodorsal shield, lateral margins of hysteronotal shield without incisions, opisthosomal lobes with lateral membrane, legs IV not extending to level of setae *h2*	***Timalinyssus actinodurae***
–	Prodorsal shield not fused with scapular shields, setae *se* on striated tegument, lateral margins with deep incisions between *e2* and *f2* setae, opisthosomal lobes without lateral membrane, legs IV with ambulacral discs extending to level of setae *h2*	***Timalinyssus wahlangi* sp. n.**

## Supplementary Material

XML Treatment for Timalinyssus wahlangi
